# Joint modeling on serum creatinine and time to end stage of renal disease for chronic kidney disease patients under treatment at the University of Gondar Referral Hospital

**DOI:** 10.1002/hsr2.1563

**Published:** 2023-09-17

**Authors:** Samson Belay, Dessie Melese, Kasim Muhammed

**Affiliations:** ^1^ Department of Statistics, College of Natural and Computational Science University of Gondar Gondar Ethiopia; ^2^ St. Paul's Hospital Millennium Medical College School of Public Health Addis Ababa Ethiopia

**Keywords:** chronic kidney disease, end stage of renal disease, joint model, serum creatinine

## Abstract

**Background and Aims:**

Chronic kidney disease (CKD) is a major health problem worldwide. The general objective of this study is to identify the joint factors of serum creatinine (SCr) and time to end stage of renal disease (ESRD) for CKD patients under treatment at University of Gondar Referral Hospital (UOGRH).

**Methods:**

A retrospective cohort study was conducted. The collected information was secondary data type obtained from 311 CKD patient's medical charts in the UOGRH from September 2019 to January 2022 G.C. Joint modeling analysis contained a linear mixed model for SCr and the Cox‐PH model for time to ESRD of CKD patients under treatment was used.

**Result:**

From the total of 311 patients, 104 (33.4%) patients were developed the ESRD, while the other 207 (66.6%) were censored patients. In the longitudinal submodel, the variable sex, age, electrolyte, visit time, anemia, diabetes mellitus, chronic heart disease, hypertension, and hepatitis have a significant effect on the variable SCr. In survival process, anemia (HR = 2.53, *p* = < 0.001), diabetes mellitus (HR = 2.206, *p* = < 0.0047), chronic heart disease (HR = 2.83, *p* = < 0.0011), HIV (HR = 2.778, *p* = < 0.0045), hypertension (HR = 2.616, *p* = < 0.001), and hepatitis (HR = 4.4, *p* = < 0.0074) have a significant effect on the variable ESRD. On the basis of the result of the joint model, the variable anemia, diabetes mellitus, chronic heart disease, hypertension, and hepatitis were common significant factors.

**Conclusion:**

The majority of patients 207 (66.6%) of CKD patients were censored. On the basis of the smaller information criteria value and the significance association value, the joint model better fits the data. In the joint model, the variable anemia, diabetes mellitus, chronic heart disease, hypertension, and hepatitis were common factors of two responses, and also concluded that the rate of progression of longitudinal measure SCr decreased over time.

**Abbreviations**
AIC: Akaki Information Criteria
BIC: Bayesian Information Criteria
BMI: Body Mass Index
CKD: chronic kidney disease
ESRD: end stage of renal disease
GFR: glomerular filtration rate
HD: hemodialysis
NDT: Neuro Developmental Technique
PH: proportional hazard
RRT: renal replacement therapy
SCr: serum creatinine
UOGRH: University of Gondar Referral Hospital
WHO: World Health Organization

## 1 BACKROUND

Chronic kidney disease (CKD) is a significant health problem that can result in the end stage of renal disease (ESRD).^1^ CKD is more significant for developing countries, which now face the double load of communicable diseases and rising problems of noncommunicable diseases, such as obesity, diabetes, and hypertension.^2^

Globally, CKD has been identified as a major public health issue in the world. The number of people with ESRD who need renal replacement therapy is expected to be between 4.902 and 7.083 million. The expected incidence of CKD was 13.4% (11.7%–15.1%). Global burden of disease and death is directly impacted by CKD through its effect on cardiovascular risk and ESRD.^3^

In sub‐Saharan Africa (SSA), adults are estimated to have a 13.9% prevalence of CKD. Hypertension, diabetes mellitus, glomerulonephritis, and HIV infection are the main causes of CKD in Africa. The prevalence of CKD ranges from 5.6% to 27.3% in those with HIV infection, from 14.1% to 32.6% in those with diabetes mellitus, and from 15% to 48% in those with hypertension. Glomerulonephritis affects up to 33% of African individuals with ESRD. Glomerulonephritis and congenital abnormalities of the kidneys and urinary tract are the key sources of CKD in kids in Africa. The estimated prevalence of ESRD attributable to diabetes and hypertension in SSA is 239 per million people, although only 1.5% of patients receive renal replacement treatment.^4^

In Ethiopia, a study was done on the prevalence of CKD and its contributing factors. Thorough investigation and meta‐analysis patients with CKD had a notably high prevalence of the chronic renal disease. A substantial correlation exists between CKD and hypertension. In Ethiopia, the prevalence of CKD is estimated to be 21.71% (95% confidence interval [CI]: 17.67, 25.74). Among patients with chronic illnesses, Oromia had the highest frequency of CKD (32.55%; 95% CI: 19.91, 45.19). Compared with people with no hypertension, people with hypertension have an increased risk of developing CKD.^5^

In Northwest Amara, the study was conducted to evaluate the incidence of CKD and its risk variables among adult hypertension patients. Between July and August 2020, 581 adult hypertension patients in a chronic follow‐up clinic at referral hospitals in Northwest Amara Ethiopia were the subjects of a facility‐based cross‐sectional study. The sample in the study was chosen by systematic random sampling. A total of 17.6% of patients with hypertension were found to have CKD. Regarding contributing causes, dyslipidemia, proteinuria, concomitant illness, serum creatinine (SCr) greater than 0.9 mg/dL, hypertension lasting longer than 10 years, and diastolic blood pressure more than 90 mmHg are all connected to the development of CKD in hypertensive individuals.^6^

A separate analysis of longitudinal and survival data, linear mixed effects models for longitudinal data, and semiparametric models for survival data was performed. However, their separate analysis is inappropriate when the longitudinal variable is correlated with time to event. Joint models of longitudinal and survival data and include complete evidence at the same time and provide effective and efficient conclusions. However, by separate modeling, the associations between the two outcomes cannot be studied. SCr is measured at dissimilar times for every patient, the SCr changes from time to time for every patient, and also there was a considerable difference in the SCr fluctuations variability among subjects. The general objective of this study is to identify the joint factors of SCr and time to ESRD for CKD patients under treatment at University of Gondar Referral Hospital (UOGRH).

The study enables the government to fulfill the necessary materials and infrastructures for the hospitalization of CKD patients until recovery from the ESRD. It also enables health professionals to give special attention to the patients who recorded very high SCr. It also enables clinicians to increase the responsiveness of society about influences that increase the chance of risk of ESRD from the treatment of CKD patients. The outcome of this study also provides evidence for public health practitioners and stakeholders who are working in the areas of giving attention, care, and handling CKD patients by modeling disease evolution to understand CKD prognosis.

## 2 METHODS

### 2.1 Data collection procedure and source of data

A secondary data collection process was used by reviewing the patient's chart and follow‐up cards on a CKD patient, who was treated in the medical ward section at UOGRH from September 2019 to January 2022. The data were collected from the medical chart of CKD patients at UOGRH from which necessary attributes have been obtained to identify the most important determinants of SCr CKD patients. Laboratory tests would be performed every 2 months, and both the longitudinal and survival data were extracted from the patient's chart which contains sociodemographic and clinical data of all renal failure patients who met the inclusion criteria. Thus, in this study, we have used secondary data obtained from CKD patients' follow‐up charts at UOGRH.

### 2.2 Study area and period

This study was conducted at UOGRH, Amhara Region, and Northern Ethiopia on CKD patients who started treatment on kidney dialysis from September 2019 to January 2022.

### 2.3 Study design

In this study, we have got information through a retrospective cohort study design where mainly joint longitudinal and survival modeling have considered identifying novel predictors.

### 2.4 Study population

All CKD patients whose ages were 16 years and above were treated on kidney dialysis follow‐up from September 2019 up to January 2022 at UOGRH.

### 2.5 Eligibility criteria

#### 2.5.1 Inclusion criteria

The study population includes all CKD patients who attended three follow‐up visits and above in the time interval of September 2019–January 2022 G.C. at the UOGRH.

#### 2.5.2 Exclusion criteria

CKD patients whose follow‐up time is less than three in the time interval of September 2019–January 2022 G.C., incomplete medical records, and all CKD patients who start their treatment before September 2019 and after January 2022 G.C. at UOGRH is excluded from this investigation.

### 2.6 Study variables

#### 2.6.1 Response variables

The continuous longitudinal SCr and time up to the complication of ESKD in patients would be the two response variables considered in this study. The continuous longitudinal outcome variable SCr was measured in mg/dL. SCr is the concentration of a compound known as creatinine in the blood or urine.^7^ The survival outcome variable would be the time at an event of clinical interest (ESRD) of CKD occurs from a defined origin. The patient transferring to a prior hospital with a good status, loss to follow‐up, death, and the study period ending in the study region were all considered as censoring in this study.

#### 2.6.2 Independent variables

In this study, predictor variables expected to be associated with repeated measures of SCr and time to ESRD of CKD patients were either sociodemographic variables, such as sex, age, residence, weight behavioral variable smoking status, and clinical variables, such as HIV, diabetes mellitus, hypertension, anemia, chronic heart disease, urine output, hepatitis, respiratory rate, and electrolyte (potassium).

### 2.7 Method of data analysis

*In descriptive statistics*, a frequency distribution table would be used to summarize the distribution of outcomes by different levels of covariates and response variables, graphs were used for Kaplan–Meier (KM) survival, and a longitudinal distribution chart would be used to summarize the distribution of SCr level frequency in each response variable category. Various socioeconomic variables and clinical factors of CKD were considered and analyzed using descriptive statistics through a considerate and detailed univariate description of the data. This contains exploratory data analysis to use an appropriate statistical model for the data. Hence, individual profile plot, mean profile plot, the KM curve, and normal Q–Q plot were performed.

In this study, we considered three different models, which mean that the linear mixed effect model (LMM) for the longitudinal SCr, the Cox proportional hazard (PH) model for time to ESRD, and a joint model for SCr and time to ESRD of CKD patients linked by shared random effects.

### 2.8 Linear mixed effect model

LMM is commonly used in which random effects include between‐subject variations and within‐subject relationships in the data. In marginal models, the mean structure and the correlation structure are modeled separately without distribution assumptions for the data while in the transitional models, the within‐subject relationship is modeled via Markov structures. Mixed models extend classical linear regression models by containing random or subject‐specific effects next to the fixed effects in the structure for the mean. The random effects not only determine the correlation structure between observations on the same subject, but they also take account of heterogeneity among subjects, due to unobserved characteristics.

### 2.9 Cox‐PH model

The basic model for survival data is the PH model, planned by Cox. This is used to determine which combination of the combination independent variables affects the form of the hazard of event and also to get an estimation of the hazard function itself for different. From this, an evaluation of the survivor functions and from now the median survival time can be got. While the model is based on the assumption of PHs, no specific form of the probability distribution is assumed for the survival times. The model is therefore referred to as a semiparametric model.

### 2.10 Joint model analysis for longitudinal and survival data

In the joint model of longitudinal and survival data effect of the longitudinal outcome (SCr) on time to ESRD for CKD patients, predictors that affect the two responses simultaneously and the association between longitudinal (SCr) and time to event (time to ESRD) were identified. The result of the joint model would be obtained by joining the selected random intercept and slope effect from the linear mixed model and the Cox‐PH model. If the test for the univariable analysis has a *p* value less than or equal to 0.25 predictors were included in the multivariable model.

## 3 RESULT

### 3.1 Descriptive statistics

In this study, sociodemographic, behavioral, and clinical variables were included that were recorded from 311 CKD at UOGRH during the period of September 2019–January 2022. The study considered two response variables: these are longitudinal measures of SCr and survival time to ESRD. The longitudinal response is the number of SCr in mg/dL of CKD patients, which were measured approximately every 2 months; at the first entry, and again at 2‐, 4‐, 6‐, 8‐, 10‐, 12‐, 14‐, 16‐, 18‐, 20‐, 22‐, and 24‐month visits.

The survival interest of the study was time to ESRD. Thus, based on the data obtained from UOGRH, out of 311 patients, 104 (33.4%) patients developed ESRD, while the other 207 (66.6%) were censored patients.

Table 1 shows the descriptive result of predictor variables of CKD patients. Out of a sample of 311 patients, 175 (56.27%) were males and the other 136 (43.73%) were female. Similarly, the percentage of event occurrence was high in males 60 (19.29%) as compared with females 44 (14.15%). A total of 225 (72.35%) of CKD patients were residents in rural areas. According to the smoking status, the majority of patients 235 (75.57%) were not smokers during the study and 76 (24.43%) were smokers during the study period. The percentage of event occurrence (ESRD) was higher for those CKD patients who have HIV 31 (9.97%), diabetes mellitus 31 (9.97%), hypertension 52 (16.72%), and chronic heart disease 21 (6.75%) as compared with those of patient who had disease and censored. The majority of CKD patients did not have HIV 253 (81.35%), diabetes mellitus 255 (81.99%), chronic heart diseases 280 (90.03%), anemia 214 (68.81%), hypertension 208 (66.88%), and hepatitis 244 (78.46%).

**Table 1:** Frequency distribution of predictor variables for CKD patients.

		Status		
		Censored	Event	Total
Variables	Categories	207 (66.6%)	104 (33.4%)	311 (100%)
Sex of patient	Male	115 (36.98%)	60 (19.29%)	175 (56.27%)
	Female	92 (29.58%)	44 (14.15%)	136 (43.73%)
Residence	Rural	148 (47.59%)	77 (24.76%)	225 (72.35%)
	Urban	59 (18.97%)	27 (8.68%)	86 (27.65%)
Smoking	No	159 (51.13%)	76 (24.44%)	235 (75.57%)
	Yes	48 (15.43%)	28 (9%)	76 (24.43%)
HIV	No	180 (57.88%)	73 (23.47%)	253 (81.35%)
	Yes	27 (8.68%)	31 (9.97%)	58 (18.65%)
Diabetes mellitus	No	182 (58.52%)	73 (23.47%)	255 (81.99%)
	Yes	25 (8.04%)	31 (9.97%)	56 (18.01%)
Hypertension	No	156 (50.16%)	52 (16.72%)	208 (66.88%)
	Yes	51 (16.4%)	52 (16.72%)	103 (33.12%)
Anemia	No	153 (49.2%)	61 (19.61%)	214 (68.81%)
	Yes	54 (17.36)	43 (13.83%)	97 (31.19%)
Chronic heart disease	No	197 (63.34%)	83 (26.69%)	280 (90.03%)
	Yes	10 (3.22%)	21 (6.75%)	31 (9.97%)
Hepatitis	No	181 (58.2%)	63 (20.26%)	244 (78.46%)
	Yes	26 (8.36%)	41 (13.18%)	67 (21.54%)

Variable	Mean	SD	Minimum	Maximum
Weight (in kg)	56.73	8.96	32	80
Age of patient (in year)	48.82	18.36	16	85
Respiratory rate (breaths per minute)	19.76	13.99	14	68
Urine output (mL)	65.09	8.59	43	87
Electrolyte (mill equivalents per liter [mEq/L])	5.181	1.05	3.2	7.4

Abbreviations: CKD, chronic kidney disease; HIV, human immunodeficiency virus.

The average baseline age of CKD patients was 48.82 with a standard deviation of 18.36. The average baseline weight of CKD patients was 56.73 with a standard deviation of 8.956. The average baseline respiratory rate of CKD patients was 19.16 with a standard deviation of 13.99. Similarly, the average baseline urine output and electrolyte of CKD patients were 65.09 and 5.18 with a standard deviation of 8.58 and 1.04, respectively. The minimum and maximum baseline weights of CKD patients were 32 and 80 kg, respectively. The minimum and maximum baseline ages of CKD patients were 16 and 85 years, respectively.

### 3.2 KM estimates

**Figure 1:**
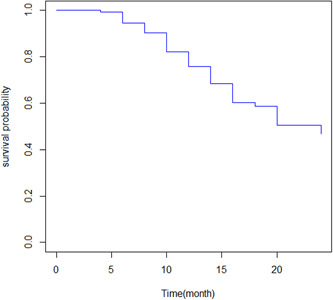
Kaplan–Meier estimate curve of chronic kidney disease patients.

Figure 1 shows that the overall KM estimate indicates that the survival probability of patients declines as the follow‐up time in the month increases. The median survival time was 24 that means 50% of individuals have survived for at least 24 months.

**Figure 2:**
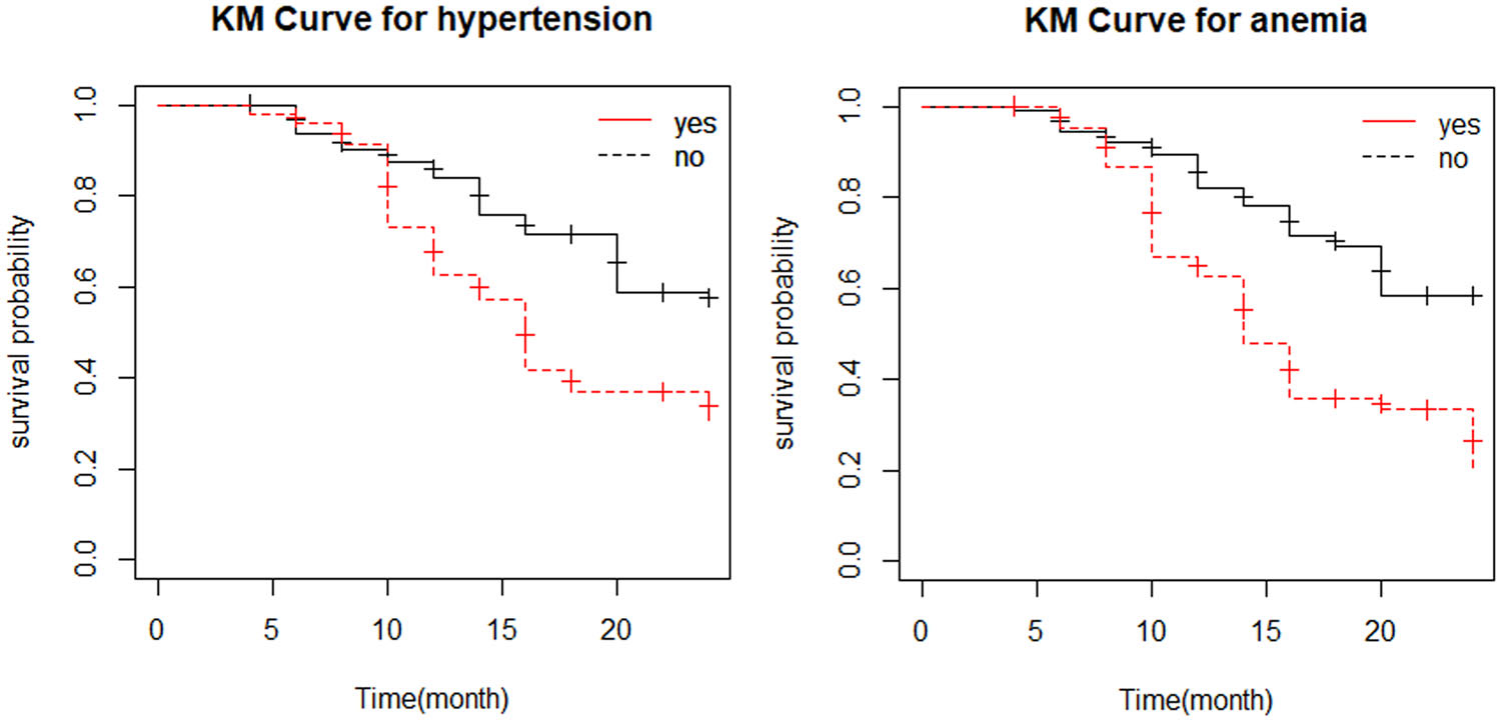
Kaplan–Meier plot of hypertension and anemia status of CKD patients. CKD, chronic kidney disease; KM, Kaplan–Meier.

The KM survival curve indicates whether there is a difference in survival time to ESRD between different categories of the variables. Figure 2 indicates the KM curve plot for the hypertension status of CKD patient indicates that the survival probability of patients who has no hypertension is high as compared with hypertensive patients at a 5% level of significance. The KM curve plot for anemia shows that the survival probability of no anemia is higher as compared with anemic patients at a 5% level of significance.

### 3.3 Cox‐PHs model for survival data

The univariable Cox‐PH regression models were fitted for every predictor to check predictors affecting time to ESRD of patients before proceeding to multivariable models. If the test for the univariable analysis has a *p* value less than or equal to 0.25, predictors were included in the multivariable model. On the basis of the univariable result variables baseline weight of patients, the presence of hypertension, HIV status, diabetes mellitus, anemia status, hepatitis, and chronic heart diseases was included for building a multivariable Cox model.

From Table 2, the multivariable analyses of Cox regression model patients who had hypertension, HIV, chronic heart disease, diabetes mellitus, hepatitis, and anemia were statistically significant and positively associated with time to ESRD for CKD patients at 5% significance level suggests that the 95% CIs of hazard ratios do not include the null value (1).

**Table 2:** Multivariable Cox proportional hazards model for selected variable of CKD patents.

Parameters	Coefficient	HR	SE (coefficient)	*Z* value	95% CI HR		*p* Value
					Lower	Upper	
Weight	−0.0187	0.98144	0.01098	−1.706	0.9605	1.003	0.088
Hepatitis (ref = no)							
Yes	1.48181	4.40091	0.27531	5.382	2.5657	7.549	<0.001
Hypertension (ref = no)							
Yes	0.96148	2.61557	0.22186	4.334	1.6932	4.040	<0.001
HIV (ref = no)							
Yes	1.02196	2.77862	0.29123	3.509	1.5701	4.917	<0.001
CHD (ref = no)							
Yes	1.03988	2.82888	0.26862	3.871	1.6710	4.789	<0.001
Debites mellitus (ref = no)							
Yes	0.79102	2.20565	0.22632	3.495	1.4155	3.437	<0.001
Anemia (ref = no)							
Yes	0.93016	2.53491	0.20088	4.630	1.7099	3.758	<0.001

Abbreviations: CHD, chronic heart disease; CI, confidence interval; CKD, chronic kidney disease; HIV, human immunodeficiency virus; HR, hazard ratio.

### 3.4 Linear mixed model for longitudinal data

On the basis of univariable analysis of the linear mixed model, the variable visit time of patient, sex, age, diabetes mellitus, chronic heart disease, status of anemia, hypertension, hepatitis, and electrolyte were significant candidate variables for multivariable analysis of the linear mixed model at a 0.25 level of significance.

The above Table 3 result showed that the novel predictor variable visit time of patient, sex, age, diabetes mellitus, chronic heart disease, status of anemia, hypertension, hepatitis, and electrolyte were significantly associated with SCr at a 5% level of significance.

**Table 3:** Result of the multivariable linear mixed model for CKD patient's parameter estimation.

Fixed effects: Parameter						95% CI	
	Estimate	SE	DF	*t* Value	*p* Value	Lower	Upper
(Intercept)	7.6549	0.4805	1763	15.9770	<0.001	6.7175	8.5924
Sex (female)	−1.8392	0.2564	308	−7.1924	<0.001	−2.3411	−1.3372
Age	0.0312	0.0068	1763	4.5837	<0.001	0.0179	0.0446
Anemia (yes)	0.6762	0.2803	1763	2.5133	<0.001	0.1497	1.2027
Hypertension (yes)	0.7379	0.7471	1763	2.7412	0.0062	0.2112	1.2645
Hepatitis (yes)	2.1218	0.3973	308	2.8349	0.0049	0.6526	3.5910
Chronic heart disease (yes)	0.8587	0.3455	1763	2.2484	0.025	0.1114	1.6060
Debites mallitus (yes)	0.8064	0.2709	1763	2.3584	0.019	0.1374	1.4754
Electrolyte	0.0864	0.0355	1763	2.4212	0.016	0.0165	0.1563
Visit time	−0.1012	0.0322	1763	−3.1689	0.0016	−0.1638	−0.0387

Random effect: Parameter			95% CI (SD)	
	Estimate	SE	Lower	Upper
Intercept ($\mathrm{stdv}(b_{0i})$)	3.665	3.665	3.345	4.007
Visit time ($\mathrm{stdv}(b_{1i})$)	0.526	1.055	0.4803	0.5766
Residual	2.302	2.302	2.255	2.351
Corr($b_{0i},b_{1i}$)	−0.822	−0.816	−0.859	−0.7756

Abbreviations: CI, confidence interval; CKD, chronic kidney disease; DF, degrees of freedom.

The result of Table 4 shows the joint modeling for the longitudinal and survival processes. The result showed that the independent variable visit time of patient, sex, age, diabetes mellitus, chronic heart disease, and status of anemia, hypertension, hepatitis, and electrolyte were statistically significant associated with SCr at a 5% level of significance in the longitudinal submodel. Also, hypertension, HIV, chronic heart disease, hepatitis, diabetes mellitus, and anemia were significantly associated with time to ESRD for CKD patients at a 5% level of significance in the survival submodel. Therefore, the predictor hypertension, chronic heart disease, diabetes mellitus, hepatitis, and anemia were affected jointly by the two response variables SCr and time to ESRD. The association parameter (*α*) indicates the association between the longitudinal measurement and the survival parts of the data. There is a relationship between longitudinal and survival outcome since the estimated parameter (*α*) in the survival submodel under joint model analysis was significant and different from zero.

**Table 4:** Result of parameter estimation of the joint model of SCr and time to ESRD.

*Longitudinal process*						
Parameters	Estimate	SE	*Z* value	*p* Value	95% CI	
					Lower	Upper
Intercept	7.6195	0.4791	15.8560	<0.001	6.6776	8.5613
Sex (female)	−1.8299	0.2557	−7.1366	<0.001	−2.3324	−1.3273
Age	0.0316	0.0068	4.6348	<0.001	0.0182	0.0449
Hypertension (yes)	0.7220	0.2692	2.5759	0.010	0.1726	1.2714
Hepatitis (yes)	2.1344	0.7485	2.8569	0.0043	0.6701	3.5987
Chronic heart disease (yes)	0.8288	0.3819	2.0860	0.037	0.0501	1.6075
Diabetes mellitus (yes)	0.7559	0.3419	2.1882	0.029	0.0788	1.4330
Anemia (yes)	0.6687	0.2691	2.4688	0.014	0.1378	1.1995
Electrolyte	0.0904	0.0357	2.5470	0.011	0.02083	0.1599
Visit time	−0.0978	0.0319	−3.0346	0.0024	−0.1609	−0.0346

**Random effects**	**SD**	**Variance**				
Intercept ($b_{0i}$)	3.6613	13.405				
Visit time ($b_{1i}$)	0.5316	0.283				
Residual	2.3003	2.691				
Corr($b_{0i}$, $b_{1i}$)	−0.8216					

*Event process*							
**Parameters**	**Estimate**	**SE**	***Z* Value**	***p* Value**	**HR**	**95% CI**	
						**Lower**	**Upper**
Intercept	4.5069	0.4668	9.6550	<0.001	90.017	3.5920	5.42183
Weight	0.0089	0.0052	1.7269	0.084	1.0089	0.8837	1.01899
Hepatitis (yes)	0.8015	0.1444	5.5515	<0.001	2.2288	1.5185	2.08447
HIV (yes)	0.3465	0.1516	2.2852	0.022	1.4141	1.0493	1.64363
Hypertension (yes)	0.4204	0.1120	3.7518	0.001	1.5225	1.2007	1.63999
Chronic heart disease (yes)	0.3321	0.1362	2.4388	0.015	1.3938	1.06521	1.5990
Diabetes mellitus (yes)	0.2889	0.1127	2.5631	0.010	1.3349	1.06798	1.5098
Anemia (yes)	0.2462	0.1029	2.3918	0.017	1.2791	1.0444	1.4479
Association	0.1300	0.0290	4.4881	<0.001	1.1388	1.0732	1.2867
AIC = 8744.49		BIC = 8829.10		log_Lik = − 4357.24			

Abbreviations: AIC, Akaki Information Criteria; BIC, Bayesian Information Criteria; CI, confidence interval; ESRD, end stage of renal disease; HIV, human immunodeficiency virus; SCr, serum creatinine.

### 3.5 Interpretation of the result

From Tables 3 and 4, we have seen that the random effect for separate and joint model analysis showed that the variation of the random intercepts was greater than the random slopes in both models; this implies that there is a greater baseline difference. The two model output also shows the correlation coefficient between the random intercept and the random slope, which means that the slope of visit time and intercept are predicted since the correlation is different from zero. Therefore, in the joint model, the variability between patients in intercept was 13.405, the variability between patients in slope was 0.283, and the correlation between intercepts and slopes was −0.8216, which shows that there is a negative correlation between intercept and slope of linear time and the variability within patients were 2.691.

Table 4 also showed the joint model of longitudinal SCr and time to ESRD of CKD patients from treatment. The interpretation of the parameter is given according to the joint result longitudinal submodel and the survival submodel. In the longitudinal process, the esteemed coefficient of fixed effect intercept was 7.6195; which means that the average SCr of the CKD patients was 7.6195 at baseline time the other variables kept constant (*p* < 0.001).

The average SCr of CKD patients who had hepatitis was significantly increased by 2.134 mg/dL (*p* < 0.0043) compared with the patient who had no hepatitis by keeping all other variables constant. In this study, visit time had a significant negative effect on SCr from the longitudinal process. If the patients visit time increased by a unit, the average SCr of CKD patients is reduced by 0.0978 (*p* < 0.0024) keeping all other variables constant. This implies that the patients with more visit time made a particular decrease in SCr. The average SCr of CKD patients who had hypertension was significantly higher by 0.722 mg/dL (*p* = 0.010) compared with the patients who had no hypertension by keeping all other variables constant.

The mean SCr of CKD patients who had chronic heart disease was significantly higher by 0.8288 mg/dL (*p* = 0.037) compared with the patients who had no chronic heart disease by keeping all other variables constant. The mean SCr of CKD patients who had diabetes mellitus was significantly higher by 0.7559 mg/dL (*p* = 0.029) compared with the patients who had no diabetes mellitus by keeping all other variables constant. The average SCr of CKD patients who had anemia was significantly higher by 0.6687 mg/dL (*p* = 0.014) compared with the patients who had no anemia by keeping all other variables constant.

For a unit increased in age the mean SCr of CKD patients was significantly increased by 0.0316 mg/dL (*p* = 0.032) by keeping all other variables constant. For a unit increase in electrolyte, the mean SCr of CKD patients was significantly increased by 0.0904 mg/dL by keeping all other variables constant by keeping all other variables constant. Similarly, the average SCr of CKD patients decreased by 1.829 mg/dL for females as compared with male patients by keeping all other variables constant.

### 3.6 Intraclass correlation

$$\text{Intra class correlation}\;=\delta_{\pi }^{2}/(\delta_{\pi }^{2}+\delta_{e}^{2})=3.665^{2}/(3.665^{2}+2.302^{2})=0.71.$$

The proportion of (unexplained) variation in the dependent variable due to subjects is 71%.

In the survival submodel, the estimated hazard ratio of ESRD for CKD patients with hepatitis was HR = 2.228, *p* = < 0.001) This indicates that the hazard of ESRD for CKD patients with hepatitis was 2.23 times higher as compared with no hepatitis CKD patients, keeping all other variables constant. The estimated hazard ratio of ESRD for CKD patients with HIV was HR = 1.4141, *p* = < 0.022) This indicates that the hazard of ESRD for CKD patients with HIV was 1.4141 times higher as compared with no HIV CKD patients, keeping all other variables constant.

The calculated hazard proportion of ESRD for CKD patients with hypertension was HR = 1.5225, *p* = < 0.001. This indicates that the hazard of ESRD for CKD patients with hypertension was 1.5225 times higher as compared with no hypertension CKD patients, keeping all other variables constant. The calculated hazard proportion of ESRD for CKD patients with chronic heart disease was HR = 1.3938, *p* = < 0.015. This indicates that the hazard of ESRD for CKD patients with chronic heart disease was 1.3938 times higher as compared with no chronic heart disease CKD patients, keeping all other variables constant.

The calculated hazard proportion of ESRD for CKD patients with diabetes mellitus was HR = 1.3349, *p* = < 0.010. This indicates that the hazard of ESRD for CKD patients with diabetes mellitus was 1.3349 times higher as compared with no diabetes mellitus CKD patients, keeping all other variables constant. The calculated hazard proportion of ESRD for CKD patients with anemia was HR = 1.2791, *p* = < 0.017. This indicates that the hazard of ESRD for CKD patients with anemia was 1.2791 times higher as compared with no anemia CKD patients, keeping all other variables constant.

The estimation of the association parameter (*α*) = 0.13 (*p* = < 001) shows that there is an indication of a positive relationship between SCr and risk of ESRD for CKD patients, which means a unit increase of SCr the risk of ESRD increased by HR = 1.1388, the result showed that the higher value of SCr was associated with the higher risk of ESRD for CKD patients in UOGRH.

## 4 DISCUSSION

The general objective of this investigation is to identify the joint factors of SCr and time to ESRD for CKD patients under treatment at UOGRH from September 2019 to January 2022. In this investigation, we applied three different models: the LMM for a longitudinal measure of SCr, the Cox‐PH ratio for survival time‐event outcomes, and the joint model for the two responses together. In the separate analysis, different assumptions were checked for longitudinal SCr and for the survival outcomes. The main challenge of the study was limited information on important predictors. Since the data were a secondary data type extracted from the patient medical charts. The other limitation of the study was the lack of enough literature in our country as well as the study area related to the study, therefore the references are more to other country outcomes.

The main contributor factors of CKD that leads to an increase in the risk of ESRD were hypertension, diabetes, and anemia. These findings were supported by Podkowińska and Formanowicz^8^ and Düsing.^9^ This is due to a patient with uncontrolled diabetes, hypertension, or both who can easily and quickly develop ESRD.

Age has a significant effect on the longitudinal measures of SCr. When the age of patients increases, the SCr measures increase for CKD patients. These results are supported by Düsing^9^ and Thomas et al.^10^ This means that higher levels of SCr are found to be increased age of patients which leads to more exposed to ESRD.

According to the results of this study, visit time had a significant negative effect on SCr suggesting that SCr levels significantly decreased over time. The patients appeared to have improved kidney function over time, as evidenced by the negative slope of the SCr level. This was in line with the results of the study conducted by Zhang et al.^7^ and Maraghi et al.^11^

In this research, there was an association between sex and SCr values indicating that males develop CKD more than females so sex is the most significant factor for CKD. This study was in line with the result of the study conducted by Zhang et al.^7^, ^12^ and Maraghi et al.,^11^ may be a result of men living unhealthier lives, the protective benefits of estrogens, or the harmful effects of testosterone. It could result from an imbalance of sex hormones. The average SCr of CKD patients who had hypertension, chronic heart disease, and diabetes mellitus was increased this finding was lined with the study done by Zhang et al.^12^ The average SCr of CKD patients who had diabetes mellitus was significantly higher as compared with none diabetic patients this finding was lined with the study done by Bash et al.^13^ This implies that patients with diabetes are more likely to acquire ESRD. The damage caused by vessel microangiopathy is the key problem associated with the rise in cardiovascular events. Diabetes‐related kidney impairment may serve as a warning sign for cardiac illnesses, which could be exacerbated if diabetic retinopathy is present.

The hazard of ESRD for CKD patients with diabetes mellitus and hypertension was higher as compared with nondiabetic and nonhypertensive CKD patients this result of our study was supported by the study report conducted by Zhang et al.,^7^ Düsing,^9^ and Zoccali et al.^14^ Since glomerulosclerosis and kidney function loss are caused by glomerulosclerosis, which is transmitted to intraglomerular capillary pressure by hypertension, a varied risk of reduced renal function has been described among hypertensive individuals. The hazard of ESRD for CKD anemic patients was higher as compared with nonanemic CKD patients. Anemia in CKD is caused by multiple factors. The endogenous erythropoietin (EPO) level gradually decreasing has traditionally been thought to play a major impact. However additional causes of anemia in CKD patients have also been identified. These included absolute iron deficiency brought on by blood loss or poo iron absorption, inefficient use of iron stores brought on by elevated hepcidin levels, systemic inflammation brought on by CKD and associated comorbidities, a decreased ability of the marrow to respond to EPO because of uremic toxins, and a short red cell life span. Similarly, patients with chronic communicable diseases such as HIV and viral hepatitis are at higher risk of ESRD for CKD this is due to the elevated risk of incident CKD and ESRD was linked to positive hepatitis B surface antigen serology. This finding of our study was lined with the study done by Zoccali et al.^14^

The median survival time of this study was 24 months which indicates that 50% of people have lived for at least 24 months and also suggests that higher SCr leads to risk for ESRD. This finding of the study lined with Ferreira et al.,^15^ which suggested that the median survival time was 4.92 years which indicates that 50% of people have lived for at least 4.92 years. Early on in ESRD treatment, survival rates are still low. Hypertension, diabetes, and anemia appear to be the main drivers of CKD which increase the SCr of patients this finding of the result lined with the result obtained by Diez Roux et al.^16^

## 5 CONCLUSION

The study investigates and identifies the factors that are associated with longitudinal measures of SCr and time to ESRD from the treatment of CKD patients based on the data obtained from UOGRH on the period of September 2019–January 2022 G.C.

The variable sex, age, electrolyte, visit time, anemia, diabetes mellitus, chronic heart disease, hypertension, and hepatitis have a significant effect on the longitudinal variable SCr at a 5% level of significance. Among them, visit time and sex (female) have a negative association with SCr, while the remaining age, electrolyte, anemia, diabetes mellitus, hepatitis, chronic heart disease, and hypertension have a positive significant effect on the variable SCr. In the survival process, anemia, diabetes mellitus, chronic heart disease, HIV, hypertension, and hepatitis have a significant and positive association effect on the variable risk of ESRD at a 5% level of significance.

On the basis of the result of the joint model, the variable anemia, diabetes mellitus, chronic heart disease, hypertension, and hepatitis were common factors for a longitudinal measure of SCr and time to ESRD. The variable longitudinal measure of SCr has an association with time to ESRD; hence the association parameter is different from zero. The rate of progression of SCr decreases over time hence visit time had a negative association with SCr in the longitudinal model. The mean profile plot indicated that the average SCr decreased as the visit time increased the rate of progression of SCr decreased over time.

## LIMITATION OF THE STUDY

The main challenge of the study was limited information on important predictors. Since the data were a secondary data type extracted from the patient medical chart basic sociodemographic and behavioral variables such as marital status, religion, family history of CKD, physical exercise, education level, and alcohol use were unable to obtain from the medical chart. Thus, the variables are not included in the study. The other limitation of the study was the lack of enough literature in our country as well as the study area related to the study, therefore the reference is more to other country outcomes.

## AUTHOR CONTRIBUTIONS

**Samson Belay**: Formal analysis; investigation; methodology; software; validation; visualization. **Dessie Melese**: Formal analysis; software; supervision; validation; visualization; writing—original draft; writing—review and editing. **Kasim Muhammed**: Conceptualization; supervision; validation.

## CONFLICT OF INTEREST STATEMENT

The authors declare no conflict of interest.

## ETHICS STATEMENT

The ethical clearance approval letter was obtained from the Ethical Approval Committee of the University of Gondar, College of Natural and Computational Science. According to the rules and principles of the ethical approval of the University of Gondar, the name of the subject will not be extracted to insure the privacy of the patients, and confidentiality was maintained through the data collection and analysis process. To collect the data permission was obtained from UOGRH.

## TRANSPARENCY STATEMENT

The lead author Dessie Melese affirms that this manuscript is an honest, accurate, and transparent account of the study being reported; that no important aspects of the study have been omitted; and that any discrepancies from the study as planned (and, if relevant, registered) have been explained.

## Data Availability

The data used to support the findings of this study are available from the corresponding author upon request.
